# Long-term health outcomes of allogeneic hematopoietic stem cell transplantation

**DOI:** 10.3389/fonc.2023.1175794

**Published:** 2023-04-12

**Authors:** Amar H. Kelkar, Joseph H. Antin, Roman M. Shapiro

**Affiliations:** ^1^ Division of Stem Cell Transplantation and Cellular Therapies, Department of Medical Oncology, Dana-Farber Cancer Institute, Boston, MA, United States; ^2^ Department of Medicine, Harvard Medical School, Boston, MA, United States

**Keywords:** hematopoietic cell transplantation, long-term complications, survivorship, chronic graft-versus-host disease, chronic health conditions

## Abstract

**Background:**

Fifty years of hematopoietic cell transplantation (HCT) has ushered in an exciting era of cellular therapy and has led to enormous progress in improving the outcomes of patients with both malignant and non-malignant hematologic disease. As the survival of transplanted patients has increased, so has the recognition of long-term complications related to this therapy.

**Purpose:**

The goal of this review is to highlight some of the most common long-term complications of HCT.

**Data sources:**

To this end, we have conducted a review of the published literature on the long-term complications of HCT encompassing the past 50 years.

**Study selection:**

We have endeavored to include long-term complications reported in research articles, case series and case reports, reviews, and abstracts. We have focused primarily on adult allogeneic HCT, but have included some data from studies of pediatric allogeneic HCT as well. We have also prioritized the literature published in the last 15 years.

**Data extraction:**

Key data supporting the onset and prevalence of the most common long-term complications was extracted.

**Limitations:**

While the list of long-term complications extracted and reported was comprehensive, it was not exhaustive.

**Conclusions:**

We have endeavored to highlight some of the most common long-term complications of HCT, the recognition and management of which constitutes an important part of HCT survivorship care.

## 1 Introduction

It has been 50 years since the first donor-derived bone marrow was transplanted into a patient, a remarkable achievement at a time when there was little to no understanding of human leukocyte antigen (HLA) typing and matching, graft-versus-host disease (GVHD), and infections occurring in the context of immune compromise (1). With the benefit of hindsight, it is not surprising that recipient mortality was common and quick following allogeneic hematopoietic cell transplantation (HCT). Nevertheless, it was the dawn of the era of cellular therapies–an era marked by novel immunotherapies, including checkpoint inhibitors, donor lymphocyte infusion, CAR-T cell therapies, and NK cell therapies.

Survival after HCT has been improving over time. Registry data have demonstrated that survival without relapse at 2 years after HCT, conferred long-term survival approaching that of age-matched controls (2), which improved on prior longitudinal studies (3). While relapse remains the predominant cause of mortality beyond 2 years in patients transplanted for malignant disease (2), there has been greater recognition of the long-term effects of HCT as life expectancy has increased. Other important causes of late mortality were related to chronic GVHD (cGVHD), infection, and secondary malignancies (2).

Long-term survivors of HCT may have numerous chronic health conditions related to the transplant. In a multicenter retrospective study of HCT survivors who lived at least 10 years after transplant, the cumulative incidence of chronic health conditions was above 70% and increased annually (4). This was significantly higher than healthy siblings. The most common long-term chronic health conditions among the survivors included coronary artery disease, auditory or visual impairment, gastrointestinal, and musculoskeletal conditions (4). However, all other organs may be affected by long-term complications of HCT. Additionally, there are concerns for telomere shortening and accelerated aging because of HCT (5, 6).

In many ways, this article reflects the successes of 50 years of transplantation, describing complications of HCT that would not be apparent without many patients attaining long-term survival. We highlight some of the most common late complications of HCT within each organ system, with their recognition and management now being an important part of HCT survivorship (**Table 1**).

**Table 1:** Summary of High-Incidence Late Effects of Allogeneic HCT.

Organ system	Late Effect^*,**^	Time Range of Onset After HCT	Cumulative Incidence	References
**Cardiovascular**	Arterial events	NR	1.5% at 5 years, 4.1% at 10 years, 12.8% at 20 years, 22.1% at 25 years; 22% at 5 years	Tichelli, Blood 2007; Auberle, Cardio-Oncology 2023
	LVEF < 45%	NR	9% at 5 years	Auberle, Cardio-Oncology 2023
	Atrial Arrhythmia	NR	7% at 5 years	Auberle, Cardio-Oncology 2023
	Ischemic Stroke	4-10 years	1-5%	DeFilipp, BBMT 2017
**Pulmonary**	Late-onset noninfectious pulmonary complications	8-12 months; 177 days	10% to 26%; 13% at 12 months	Patriarca, Curr Stem Cell Res Ther 2009; Solh, Transplantation 2011
	DAH	Median onset 177 days (range, 81–1017 days) post HCT (Solh)	5% at 12-months (95% CI 3-7%)	Solh, Transplantation 2011
	BOS/BO/BOOP	Median onset 177 days (range, 81–1017 days) post HCT (Solh)	4% at 12-months (95% CI 2-5%)	Solh, Transplantation 2011
	IPS/ARDS	Median onset 177 days (range, 81–1017 days) post HCT (Solh)	4% at 12-months (95% CI 2-6%)	Solh, Transplantation 2011
**Gastrointestinal**	GVHD causing late GI symptoms	Within 6 months of HCT	~18% of HCT patients	Sung, BBMT 2017; Flowers Blood 2015
	Liver cirrhosis	Median of 10 years after HCT, although cirrhosis has been reported to occur as early as a year post HCT	4-24% at 20 years	Inamoto, Hematologica 2017; Strasser Blood 1999
	Cancer	Mouth/pharyngeal cancer onset ~1-4 years Oral cavity cancer onset ~5 years Esophageal cancer onset ~1-4 years	32-92 per 100,000 person yearsˠ 14-100 per 100,000 person years 4-59 per 100,000 person years	Inamoto, BMT 2016
**Renal**	Renal Failure	NR	< 1% at 1 year	Vrooman, BBMT 2017
	Hemorrhagic Cystitis	NR	< 1% at 1 year	Vrooman, BBMT 2017
**Endocrine**	Diabetes	1-3 years	8-41%	Inamoto, Hematologica 2017; Shaw BBMT 2017
	Thyroid dysfunction	Subclinical compensated hypothyroidism occurs in up to 15% of patients in the first year	30% by 25 years after BMT	Inamoto, Hematologica 2017; Majhail BBMT 2012
	Osteoporosis	Bone loss occurs 6-12 months post HCT	Up to 50% of patients post BMT	Inamoto, Hematologica 2017
	Avascular necrosis	Median of 12 months post HCT	Cumulative incidence of 3-15% post BMT	Inamoto, Hematologica 2017; Bhatia Exp Rev Hem 2011; Enright H AJM 1990; Socie BJH 2003
	Gonadal dysfunction	Most onset early after conditioning, but subset develop late dysfunction after the first year	3-15% of long-term survivors	Phelan et al, TCT 2021; Buchbinder et al, BBMT 2013
**Dermatologic**	GVHD causing late skin symptoms	Median onset 4-6 months after HCT	~70% of all patients who develop GVHD	Lee, ASHed 2008; Majhail, BBMT 2012
	Skin cancer	May onset as early as 1 year, but most onset > 10 years post transplant	3-6% at 20 years	Inamoto, BMT 2016; Majhail BBMT 2012
**Neurologic**	Muscle Cramping	Median of 6 months in those who developed chronic GVHD	33-66% of patients with chronic GVHD	Kraus, PLOS ONE 2012; Bilic, BMT 2016; Lehky, TCT 2022
	Altered sensation	Median onset 6 months post HCT	~20% of patients with neuromuscular complications	Bilic, BMT 2016
	Other neuropathy	Median onset 6 months post HCT	Up to 65% at 14 months post HCT	Sostak, Neurology 2003
**Psychiatric**	Fear of Progression	<6 months	29% at ~2 years	Hefner, BMT 2014
	PTSD	<6 months	15% at ~2 years	Hefner, BMT 2014
	Depression	<6 months	27% at ~2 years; 7.7% at 10+ years	Hefner, BMT 2014; Sun, BBMT 2013
	Anxiety	<6 months	27% at ~2 years; 3.4% at 10+ years	Hefner, BMT 2014; Sun, BBMT 2013
	Sleep Disturbance	<6 months	43% at ~6 years	Bishop, JCO 2007
**Ophthalmologic**	Dry eyes	Majority within 3 months of onset of chronic GVHD, ~20% develop 3 months - 2 years of onset of chronic GVHD	40-60% in those with cGVHD	Mohty, ASH Ed 2010; Sun Y-C BBMT 2015
	Cataracts	3-4 years	> 80% at 6-10 years post BMT	Mohty, ASH Ed 2010; Inamoto BBMT 2019
	Ischemic microvascular retinopathy	Onset within 6 months post HCT	Up to 10%	Inamoto BBMT 2019
	CMV retinitis	Onset dependent on timing of CMV reactivation	5-23% in patients with CMV viremia	Inamoto BBMT 2019
**Late Infectious**	NR	NR	6.4% at 12 years; 10% at 12 years (if still on IS at 2-years)	Norkin, BBMT 2019
**Hematologic**	VTE	NR	2.4% at 5 years; 4.9% at 10 years; 7.1% at 20 years	Gangaraju, BA 2021
**Autoimmune**	Cytopenias	Early-onset: 2-8 months; Late-onset: 6-18 months	Early-onset: 1%; Late-onset: 2%	Chen, BMT 1997

*frequency of occurrence greatest 1 year or later after HCT.

**Must have incidence >baseline population risk.

ˠcumulative incidence rate after 10 years.

## 2 System-specific late effects

### 2.1 Cardiovascular complications

Late cardiac effects are an important potential complication of HCT, that can significantly affect the long-term health and quality of life of survivors. These might include cerebrovascular disease, coronary arterial disease (CAD), and peripheral arterial disease. The standardized mortality ratio (SMR) of HCT survivors with cardiovascular disease was previously reported to be 2.3-fold compared to controls (7), while a more recent study reported 4.1-fold (8).

The 5-year cumulative incidence of a late cardiac event in a large single-center retrospective study was 22%, with the most frequent events being 9% decreased left ventricular ejection fraction (LVEF) < 45%, 7% atrial arrhythmia, 7% elevated NT-pro BNP, 3% pericardial effusion, 2% CAD requiring percutaneous coronary intervention, 1% ventricular arrhythmia, and 1% pericarditis (9). Another large single-center study reported cumulative incidence of late cardiac events rising from 1.5% at 5 years to 22.1% at 25 years (10). Cumulative incidence of a first cardiovascular event at 15 years (CAD, stroke, or peripheral vascular disease) was 6% in a large European registry study (11). Across several studies, the cumulative incidence of stroke was 1-5%, occurring at a median of 4-10 years (12). One of those studies noted higher prevalence of stroke after 10 years in HCT survivors at 4.8% compared to 3.3% in the general population (13). Additionally, a multicenter prospective observational study found that 13% of HCT survivors had asymptomatic cardiac dysfunction, despite generally good health (14).

While late cardiac events are not uncommon, there is a stark difference in the cumulative incidence by age at the time of transplant (8). Cumulative incidence of cardiovascular events was 8.7% at 20-years in survivors receiving HCT prior to age 20, 20.2% if transplanted between ages 20 and 40, and 50.1% if transplanted between ages 40 and 60 (10). In a large registry study, less than 1% of patients transplanted before the age of 3 had developed congestive heart failure by 1-year after HCT (15). However, a longitudinal assessment of cardiovascular performance found significantly impaired cardiovascular function in pediatric HCT survivors (16). These data support the hypothesis that there may be an age-dependent cardiotoxicity effect that may be initially silent.

Due to the delayed onset of many of these late cardiac effects, several studies have explored risk factors, though they have not been consistent across studies. Older age was the most common risk factor across studies, with the large risk increase above the age of 40 (8, 10). By univariate analysis, prior total body irradiation (TBI) exposure alone, TBI with anthracycline exposure, and pre-transplant arterial diseases including LVEF < 45% and stroke were all risk factors (9, 14). However, the only strongly linked risk factors by multivariate analysis were combinations of traditional cardiovascular risk factors, such as arterial hypertension, dyslipidemia, and diabetes (10). Notably, 2-year incidence of hypercholesterolemia and hypertriglyceridemia were each over 70%, and were each independently associated with prior aGVHD (17). Twenty-nine percent of all survivors required statin therapy (17). At 25-years after HCT, cumulative incidence of dyslipidemia was 89% (18).

Evidence for prior chronic cGVHD as an independent risk factor for cardiac disease was mixed (9, 19). However, there is a biological rationale for cardiac cGVHD causing arterial disease. In a cadaveric study, two patients exhibited concentric luminal narrowing of coronary arteries due to intimal proliferation of foamy macrophages and new connective tissue, which resembled tissue changes seen in other forms of cGVHD (20). This differs from the typical atherosclerotic pattern seen in CAD and other arterial diseases and supports the notion that more longitudinal studies are needed, particularly with the rising age of patients undergoing HCT.

### 2.2 Noninfectious pulmonary complications

Arguably the most important and deadly late effects in HCT survivors are late-onset noninfectious pulmonary complications (LONIPC) (21–23). The SMR of survivors with LONIPCs was previously reported to be 15.1-fold (7), and 13.9-fold in a recent study (8). Cumulative incidence of LONIPCs ranges from 10% to 26%, with a median time of 8-12 months after HSCT for their development (23–25). Most survivors developed their first episode of LONIPC within 36 months of HCT (23). One-year incidence of specific complications was 5% diffuse alveolar hemorrhage, 4% bronchiolitis obliterans (BOS), and 4% idiopathic pneumonia syndrome.

A common finding across studies was the coincidence of cGVHD and LONIPCs, seen in both univariate and multivariate analyses (14, 22, 25). LONIPCs were also associated with higher non-relapse mortality and lower relapse rates (22). Other risk factors for LONIPCs, particularly BOS, included male sex, chest irradiation prior to HCT, history of lower respiratory tract infection within 100 days after HCT, use of peripheral blood stem cells for transplantation, 10% decline in 1-second forced expiratory volume (FEV1) between HCT and 100 days after HCT, bronchial abnormalities on CT at 100 days after HCT, and Black race (8, 23, 25). These findings highlight the need for close monitoring and early intervention to prevent and manage LONIPCs after HCT.

### 2.3 Gastrointestinal complications

Gastrointestinal manifestations are among the most common late effects in HCT survivors, as seen in a longitudinal study at 10 or more years (4). Cirrhosis accounted for 50% of gastrointestinal late effects. Cirrhosis has a cumulative incidence of 4-24% by 20 years and notably contributed to late mortality (26). Before the advent of routine hepatitis C (HCV) donor screening, HCV reactivation was the strongest predictor post-HCT cirrhosis. Other contributing risk factors included liver cGVHD, hepatitis B (HBV) infection, and iron overload (27, 28). Additionally, survivors treated with anti-CD20 antibodies after HCT were at increased risk for HBV reactivation and liver failure (26). However, hepatic iron overload, a common sequelae of hematologic malignancies and HCT, has not been strongly linked to cirrhosis, acute GVHD (aGVHD) or cGVHD, survival, NRM, relapse, organ failure, infections, or hepatic veno-occlusive disease (29–32).

In survivors of HCT with late gastrointestinal manifestations including nausea or vomiting, diarrhea, abdominal pain or cramping, and anorexia, investigation with endoscopy found GI GVHD as the most common etiology, followed by infections, predominantly cytomegalovirus (33). Other persistent gastrointestinal complications of HCT include xerostomia, esophageal stricture, and malnutrition are also typically associated with cGVHD (34).

Secondary gastrointestinal malignancies are an important late complication of HCT for which there must be clinical vigilance (35). Observational data suggests 40% 5-year mortality after developing secondary gastrointestinal malignancies (36). Risk for oral cavity cancers are also increased in survivors with persistent oral cGVHD, history of radiation therapy, or prolonged immune suppression (IS) > 24 months (35). Those with persistent GERD or dysphagia after HCT, or those receiving prolonged IS for cGVHD are at increased risk for esophageal cancer (35). Consideration may be given to enhanced screening for oral or esophageal malignancies in these patients. Notably, there has been insufficient evidence to suggest that colorectal cancer incidence is higher among HCT survivors, so general colorectal cancer screening guidelines should apply.

### 2.4 Renal complications

A large registry study of patients transplanted before the age of 3 over a 25-year period, found non-malignant late effects over 1-year after HCT in 30% of patients, but the frequency of renal failure severe enough to warrant renal replacement therapy was < 1% (2 of 717) (15). The frequency of hemorrhagic cystitis was also < 1% (1 of 717). In survivors of second HCT, cumulative incidence of renal failure at 2 and 10 years, respectively, for survivors transplanted before age of 18 was 1% and 4% (37). For survivors transplanted after the age of 18, 2- and 10-year cumulative incidences were 3% and 4% respectively. Another study looking at the impact of cGVHD on late effects found a 4-fold increase in renal dysfunction in patients with cGVHD compared to survivors without cGVHD (38). Notable risk factors for late renal disease in survivors include arterial hypertension, calcineurin inhibitor (CNI) use, thrombotic microangiopathy, radiation-induced obliterative endarteritis, and outlet obstruction from severe scarring due to BK virus, adenovirus, and cyclophosphamide. While long-term renal complications appear rare, we must continue to monitor for these, particularly with an increasingly older group undergoing HCT.

### 2.5 Endocrine complications

The most common late endocrinologic effects of HCT are classified as immunological or related to chronic steroid use (39, 40). Among the former, the thyroid gland and gonadal system are commonly involved. The latter tend to manifest as physiological aberrations, including diabetes, adrenal, and bone effects.

There is an increased incidence of type II diabetes among patients undergoing an allogeneic stem cell transplant, with the incidence reported to be around 30-40% (12, 26). Exposure to high-dose steroids (> 0.25 mg/kg/day) increased the likelihood of having persistent Type II diabetes at 2 years after HCT. Center for International Blood and Marrow Transplant Research (CIBMTR) guidelines recommend HbA1C screening at 3 months and every 3-6 months thereafter in survivors with ongoing risk factors, including those on systemic corticosteroids (12). Exposure to high doses of corticosteroids for prolonged periods also increases risk for adrenal insufficiency, which was estimated to affect 13% of HCT survivors (26).

While prolonged corticosteroid use also results in the development of bone loss, other risk factors are also involved, including the use of CNIs, radiation, and the development of gonadal failure (26). It is estimated that osteopenia occurs in 50% of adult survivors at 4-6 years after HCT, with the most significant bone loss occurring during the first 6 months (41). The cumulative incidence of osteoporosis at two years is estimated to be approximately 20%, with lifetime cumulative incidence close to 50% of survivors (26, 41). An important late bone complication of HCT is avascular necrosis, with a cumulative incidence of up to 15% in unrelated donor transplantation, and highest risk among patients with cGVHD and use of prednisone, CNIs, and MMF (26, 41).

Another common endocrinological late effect is thyroid dysfunction (40). Estimated cumulative incidence of late thyroid dysfunction is 25% of HCT survivors (26). Subclinical compensated hypothyroidism occurs in up to 15% within the first year (42). Overt hypothyroidism is reported to present 2-5 years after conditioning, particularly after TBI (7, 28).

Gonadal dysfunction is also very common after HCT (42). Laboratory screening for gonadal dysfunction in women is typically performed at 1 year after HCT, with corresponding evaluation in men performed as is warranted by symptoms (42). In men, low testosterone is commonly observed, particularly in survivors with cGVHD requiring prolonged IS and those with iron overload (43). Specific symptoms of hypogonadism include fatigue, loss of muscle mass, erectile dysfunction, and infertility. In premenopausal women, gonadal dysfunction manifests as onset of menopause and infertility, although resumption of fertility has been reported in some female survivors of HCT treated with lower intensity conditioning regimens (28). Hypergonadotrophic hypogonadism has been strongly associated with TBI and high-dose busulfan, with the ovaries being particularly susceptible (28). Most gonadal dysfunction from conditioning chemotherapy occurs early after HCT, but has been reported to develop after the first year in 3% of HCT survivors with related donors and 10% with unrelated donors over the next 5 years (44).

### 2.6 Dermatologic complications

Dermatologic complications are very common, typically manifesting early as aGVHD or drug reactions. Late dermatologic complications are generally related to either cGVHD or secondary malignancy. From the perspective of cGVHD, the skin is among the most affected systems with earlier manifestations being lichen planus and dry papulosquamous lesions, and later manifestations being sclerotic changes, poikiloderma, and poorly healing ulcers (45). Vitiligo has been reported as a rare late effect with a 4-year cumulative incidence of 1.73% after HCT, which was significantly higher than 0.34% in matched controls without HCT (46).

Skin cancers, including basal cell carcinoma (BCC), squamous cell carcinoma (SCC), and melanoma, are particularly important late complications of transplantation. The cumulative incidences of BCC and SCC were 3-6% at 20 years (35, 42). Transplant-related risk factors for skin malignancy include myeloablative TBI and cutaneous cGVHD (35). Patients with hereditary predisposition to malignancy, such as Fanconi anemia or dyskeratosis congenita, have even higher risk (35). Routine surveillance of the skin and evaluation of any abnormal lesions that develop on the skin, especially in those with the greatest risk for secondary skin cancer, is key for prevention.

### 2.7 Neurologic complications

Neurologic, cognitive, and neuromuscular complications following HCT may be more common than reported. In a prospective evaluation of HCT, neuropathological complications including subarachnoid hemorrhage, fungal infection, and toxoplasmosis, were among the most common causes of central nervous system (CNS) pathology and contributed to high early mortality (47). While these reflected an earlier era of HCT, it nevertheless highlights the susceptibility to infectious and vascular CNS pathology in HCT survivors. A subsequent prospective study evaluated the spectrum of late HCT neurological sequelae, finding cumulative incidence of neurologic complications at a median of 14 months was 65% (48). Most cases were subacute peripheral neuropathies (PN), although a subgroup of patients had cognitive deterioration with white matter lesions. Approximately half of neurologic complications were of undefined origin, but there was an association with the prior aGVHD and prolonged use of IS, suggesting either immune-mediated etiology or medication side effects as potential contributors (48). A Canadian single-center study of neurologic complications following HCT noted a high incidence of posterior reversible encephalopathy syndrome (PRES) and seizure, with the majority occurring within the first 100 days (49).

A single-center retrospective study of neurologic HCT complications found PNs more commonly presented as late effects compared to CNS complications (50). These included mononeuropathies and polyneuropathies related to infections (e.g., HHV-6), medications (e.g., CNIs or foscarnet) or Guillan-Barre syndrome (GBS). In a prospective cohort of cGVHD patients, the majority of PNs were mixed small and large fiber neuropathies (51). The classical presentation of neuropathy in patients with cGVHD included neuropathic pain, painful myalgia, paresthesia, and sensory or motor loss (52).

A large single-center study of 3835 haploidentical HCT survivors found 40 immune-mediated neurological complications with the majority being unclassified PNs, and a subgroup meeting diagnostic criteria for GBS (53). These complications manifested as weakness, altered sensation, hyporeflexia, and pain. The majority of GBS patients could attain partial remission with a combination of IVIG, plasma exchange, and steroids. For patients with non-GBS immune-mediated neuropathies, replacement of vitamin B1 and B12 resulted in complete remission in several patients, highlighting the importance of nutritional deficiencies in PNs. In the remainder, the addition of immunomodulatory agents was required to attain some degree of remission.

Muscle cramps and myalgia are common late complications after HCT. In a cross-sectional study of 1745 patients who underwent transplantation, neuropathies were reported in 66% with cGVHD, with the majority reporting muscle cramps (54). In another retrospective study, clinical signs of polyneuropathy, including both proximal and distal limb distribution with sensory and motor deficits, were evident at a median of 6 months in survivors with cGVHD (55). Most survivors had an electrodiagnostic profile consistent predominantly with axonal neuropathy. Autonomic neuropathy was rare, consistent with other retrospective studies (52). The majority of affected patients also experienced muscle cramping for 1-10 minutes several times per day. The presence of cramping correlated negatively with axonal neuropathy, suggesting distinct pathophysiology. Over half of patients responded to magnesium supplementation.

While the aforementioned neurologic complications of HCT occur in the disease-free setting, CNS relapse of the original disease, especially acute leukemia, is a critically important potential long-term neurological complication (56). This can present as focal neurological deficit, or more subtly as myoclonus, mild paresthesia, or diplopia (57). Overt leukemia relapse in the cerebrospinal fluid has a poor prognosis, and therapeutic options are limited (58, 59). Clinical vigilance is paramount, as early recognition of a neurological abnormality may identify the disease when it is isolated to the CNS, a situation with potentially favorable outcome compared to both CNS and systemic relapse (60).

### 2.8 Psychiatric complications

Psychiatric late effects of HCT survivors have long been acknowledged but have only recently been defined and characterized. A survey study of 50-consecutive HCT survivors at a median follow-up of approximately 2-years, found that 27% had moderate or severe symptoms of anxiety and depression, with fear of progression present in 29%, and significant symptoms of PTSD present in 15% (61). Younger patients (<55) reported significantly more fear of progression. A large multicenter retrospective study using the brief symptom inventory (BSI-18) found that while the prevalence of anxiety and depression was comparable between HCT survivors and healthy siblings at a follow-up of at least 10-years post-HCT, survivors were 2.7 times more likely to report somatic distress (10.8% vs. 3.9%) (4). In that study, female gender, low annual household income (< $20,000/year), poor self-rated health status, and active cGVHD were associated with increased risk for somatic distress among HCT survivors. Another study of survivors at a median of 6-years post-HCT found that 43% reported sleep disturbance post-transplant and this was significantly higher than controls (62). These findings emphasize the importance of monitoring psychiatric side effects in allogeneic HCT survivors.

### 2.9 Ophthalmologic complications

Long-term ocular complications of transplant are common and generally fall into two categories: cGVHD-related and non GVHD-related. Dry, gritty, or painful eyes are distinctive manifestations of ocular cGVHD, and may be associated with keratoconjunctivitis sicca (34, 63, 64). The cumulative incidence of ocular cGVHD has been reported in more than 50% of patients who develop cGVHD, presenting with ocular redness, irritation, and photophobia (65).

Non-GVHD ocular late effects are less common than ocular cGVHD, but still affect a considerable proportion of HCT survivors. The most common is cataract formation, with a cumulative incidence of 46% at 15 years (41). Risk factors include TBI and long-term corticosteroids (64). Ischemic microvascular retinopathy can onset suddenly or progressively, resulting in blurred vision or loss of color vision (63, 64). Cumulative incidence is reported to be as high as 10% with onset typically within 6 months of HCT. The pathophysiology is suspected to be related to CNIs in combination with baseline cardiovascular factors contributing to vascular endothelial injury (64). Additional ocular complications, including those related to ocular infections, drug toxicities, vascular and retinal damage have also been described (64).

### 2.10 Other complications

#### 2.10.1 Late infectious complications

Long-term survivors of allogeneic stem cell transplant are at increased risk for mortality due to late severe infection, with a SMR of 52.0 (95% CI 46.9-57.3) (8). Multiple studies have suggested that there is a subgroup of HCT patients with impaired immune reconstitution 2 years after HCT, resulting in subpar responses to vaccination and increased risk for opportunistic infections (66). This could result in pneumocystis pneumonia in survivors with otherwise seemingly recovered CD4+ T cell counts, or else reactivation of herpes simplex or varicella zoster viruses after discontinuation of acyclovir prophylaxis (66). Bacterial infections remained the predominant cause of late infection-related mortality in a multicenter retrospective study among survivors disease-free at 2 years. Patients with cGVHD who were on long-term IS had higher mortality risk due to infection, with 10% 12-year cumulative incidence of fatal late infections (66).

#### 2.10.2 Hematologic complications

Non-immune hematologic late effects of HCT have only been rarely studies. One found that risk for venous thromboembolism at 2-years or more post-HCT was 7.3 times higher in survivors compared to siblings. Cumulative incidence of late VTE was 2.4% at 5 years, 4.9% at 10 years, and 7.1% at 20 years (67). This study was used to build a clinical risk rule using history of stroke, cGVHD, hypertension, sex, and stem cell source. Called BMTSS-HiGHS2, this model classifies high or low VTE risk at 2-years post-HCT.

#### 2.10.3 Autoimmune complications

There is limited data on non-GVHD late autoimmune (i.e., alloimmune) complications of HCT. A large single center study found an early-onset cold autoimmune hemolytic anemia (AIHA) in 1% (4 of 293) between 2 and 8 months after HCT and a warm AIHA in 2% (5 of 293) between 6 and 18 months (68). A case-control study also described late autoimmune cytopenias presenting at a median of 219 days post-HCT (69). Of 20 patients, 13 had autoimmune hemolytic anemia, 5 had immune thrombocytopenia, 1 had Evan’s Syndrome, and 1 had autoimmune neutropenia. Response rates to frontline therapies (primarily rituximab and corticosteroids) were only 25% and many cases were further complicated by steroid toxicity.

## 3 Conclusions

Fifty years of transplantation has seen enormous leaps in the understanding and success of this form of cellular therapy for the treatment of both malignant and non-malignant disease. Early mortality from transplantation has been largely prevented as a result of a much more sophisticated understanding of HLA, GVHD, and the potential infections that afflict a severely compromised immune system. With improved survival, HCT survivors may now have to contend with long-term complications that manifest in various organs and that may significantly impact both duration and quality of life. cGVHD was a common thread as a risk factor for many late effects, and it may accentuate the late effects of chemotherapy and radiation. Thus, the impact of declining GVHD incidence on late effects of HCT is an open question. As the field evolves, we’ll also have to identify and manage late effects in older HCT survivors, and in those undergoing novel conditioning regimens and GVHD prophylaxis.

We have endeavored to highlight the most common of the long-term HCT complications, with the understanding that our list is expected to grow as the field embarks on its next 50 years of progress. Recognition and management of these complications will continue to be an important part of HCT survivorship.

## Author contributions

All authors provided substantial contributions to the conception of the work and interpretation of data, drafted and revised the work critically for important intellectual content, provided approval for publication of the content, and agree to be accountable for all aspects of the work in ensuring that questions related to the accuracy or integrity of any part of the work are appropriately investigated and resolved.
